# Supplementary material for: The experiences of patients with peritoneal dialysis: A systematic review of qualitative evidence protocol
Source: PLoS One. 2023 Jul 19;18(7):e0288724. doi: 10.1371/journal.pone.0288724 (PMC10355407; doi:10.1371/journal.pone.0288724)
Supplement: S1 Appendix — (DOCX) [file pone.0288724.s001.docx]

**Pubmed**

#1 Peritoneal Dialysis [Mesh] OR Dialyses, Peritoneal [Title/Abstract] OR Dialysis, Peritoneal [Title/Abstract] OR Peritoneal Dialyses [Title/Abstract]

#2 Life Change Events [Mesh] OR event*, life change [Title/Abstract] OR life change event [Title/Abstract] OR life experience* [Title/Abstract] OR experience*, life [Title/Abstract] OR analysis, event history [Title/Abstract] OR analyses, event history [Title/Abstract] OR event history analyses [Title/Abstract] OR event history analysis [Title/Abstract] OR personal experience [Title/Abstract] OR Perception [Mesh] OR Sensory Processing [Title/Abstract] OR Processing, Sensory [Title/Abstract] OR Attitude [Mesh] OR Attitudes [Title/Abstract] OR Opinion*[Title/Abstract]

#3 Organizational Case Studies [Mesh] OR Case Studies, Organizational [Title/Abstract] OR Studies, Organizational Case [Title/Abstract] OR Management Case Studies [Title/Abstract] OR Case Studies, Management [Title/Abstract] OR Studies, Management Case [Title/Abstract] OR Anthropology, Cultural [Mesh] OR Cultural Anthropology [Title/Abstract] OR Material Culture [Title/Abstract] OR Culture, Material [Title/Abstract] OR Material Cultures [Title/Abstract] OR Ethnography [Title/Abstract] OR Ethnographies [Title/Abstract] OR grounded theory [Title/Abstract] OR interview [Title/Abstract] OR narrative [Title/Abstract] OR phenomenology [Title/Abstract] OR qualitative method [Title/Abstract] OR qualitative research [Title/Abstract] OR qualitative stud* [Title/Abstract] OR thematic analysis [Title/Abstract] OR focus group* [Title/Abstract] OR descriptive Research [Title/Abstract] OR discourse analysis [Title/Abstract]

#4 #1 AND #2 AND #3

**Embase**

#1 'peritoneal dialysis':ti,ab,kw OR 'dialyses, peritoneal':ti,ab,kw OR 'dialysis, peritoneal':ti,ab,kw OR 'peritoneal dialyses':ti,ab,kw

#2 'life change events':ti,ab,kw OR 'event*, life change':ti,ab,kw OR 'life change event':ti,ab,kw OR 'life experience*':ti,ab,kw OR 'experience*, life':ti,ab,kw OR 'analysis, event history':ti,ab,kw OR 'analyses, event history':ti,ab,kw OR 'event history analyses':ti,ab,kw OR 'event history analysis':ti,ab,kw OR 'personal experience':ti,ab,kw OR perception:ti,ab,kw OR 'sensory processing':ti,ab,kw OR 'processing, sensory':ti,ab,kw OR attitude*:ti,ab,kw OR opinion*:ti,ab,kw

#3 'organizational case studies':ti,ab,kw OR 'case studies, organizational':ti,ab,kw OR 'studies, organizational case':ti,ab,kw OR 'management case studies':ti,ab,kw OR 'case studies, management':ti,ab,kw OR 'studies, management case':ti,ab,kw OR 'anthropology, cultural':ti,ab,kw OR 'cultural anthropology':ti,ab,kw OR 'material culture*':ti,ab,kw OR 'culture, material':ti,ab,kw OR ethnography:ti,ab,kw OR ethnographies:ti,ab,kw OR 'grounded theory':ti,ab,kw OR interview:ti,ab,kw OR narrative:ti,ab,kw OR phenomenology:ti,ab,kw OR 'qualitative method':ti,ab,kw OR 'qualitative research':ti,ab,kw OR 'qualitative stud*':ti,ab,kw OR 'thematic analysis':ti,ab,kw OR 'focus group*':ti,ab,kw OR 'descriptive research':ti,ab,kw OR 'discourse analysis':ti,ab,kw

#4 #1 AND #2 AND #3

**PsycINFO (APA)**

SU ( Peritoneal Dialysis OR Dialyses, Peritoneal OR Dialysis, Peritoneal OR Peritoneal Dialyses ) AND SU ( Life Change Events OR event*, life change OR life change event OR life experience* OR experience*, life OR analysis, event history OR analyses, event history OR event history analyses OR event history analysis OR personal experience OR Perception OR Sensory Processing OR Processing, Sensory OR Attitude OR Attitudes OR Opinion* ) AND SU ( Organizational Case Studies OR Case Studies, Organizational OR Studies, Organizational Case OR Management Case Studies OR Case Studies, Management OR Studies, Management Case OR Anthropology, Cultural OR Cultural Anthropology OR Material Culture* OR Culture, Material OR Ethnography OR Ethnographies OR grounded theory OR interview OR narrative OR phenomenology OR qualitative method OR qualitative research OR qualitative stud* OR thematic analysis OR focus group* OR descriptive Research OR discourse analysis )

**CINAHL Complete (EBSCO)**

SU ( Peritoneal Dialysis OR Dialyses, Peritoneal OR Dialysis, Peritoneal OR Peritoneal Dialyses ) AND SU ( Life Change Events OR event*, life change OR life change event OR life experience* OR experience*, life OR analysis, event history OR analyses, event history OR event history analyses OR event history analysis OR personal experience OR Perception OR Sensory Processing OR Processing, Sensory OR Attitude OR Attitudes OR Opinion* ) AND SU ( Organizational Case Studies OR Case Studies, Organizational OR Studies, Organizational Case OR Management Case Studies OR Case Studies, Management OR Studies, Management Case OR Anthropology, Cultural OR Cultural Anthropology OR Material Culture* OR Culture, Material OR Ethnography OR Ethnographies OR grounded theory OR interview OR narrative OR phenomenology OR qualitative method OR qualitative research OR qualitative stud* OR thematic analysis OR focus group* OR descriptive Research OR discourse analysis )

**Web of Science**

TS=(( Peritoneal Dialysis OR Dialyses, Peritoneal OR Dialysis, Peritoneal OR Peritoneal Dialyses ) AND ( Life Change Events OR event*, life change OR life change event OR life experience* OR experience*, life OR analysis, event history OR analyses, event history OR event history analyses OR event history analysis OR personal experience OR Perception OR Sensory Processing OR Processing, Sensory OR Attitude OR Attitudes OR Opinion* ) AND (Organizational Case Studies OR Case Studies, Organizational OR Studies, Organizational Case OR Management Case Studies OR Case Studies, Management OR Studies, Management Case OR Anthropology, Cultural OR Cultural Anthropology OR Material Culture* OR Culture, Material OR Ethnography OR Ethnographies OR grounded theory OR interview OR narrative OR phenomenology OR qualitative method OR qualitative research OR qualitative stud* OR thematic analysis OR focus group* OR descriptive Research OR discourse analysis ))

**The Cochrane Library**

#1 (Peritoneal Dialysis OR Dialyses, Peritoneal OR Dialysis, Peritoneal OR Peritoneal Dialyses):ti,ab,kw

#2 (Life Change Events OR event*, life change OR life change event OR life experience* OR experience*, life OR analysis, event history OR analyses, event history OR event history analyses OR event history analysis OR personal experience OR Perception OR Sensory Processing OR Processing, Sensory OR Attitude OR Attitudes OR Opinion*):ti,ab,kw

#3 (Organizational Case Studies OR Case Studies, Organizational OR Studies, Organizational Case OR Management Case Studies OR Case Studies, Management OR Studies, Management Case OR Anthropology, Cultural OR Cultural Anthropology OR Material Culture* OR Culture, Material OR Ethnography OR Ethnographies OR grounded theory OR interview OR narrative OR phenomenology OR qualitative method OR qualitative research OR qualitative stud* OR thematic analysis OR focus group* OR descriptive Research OR discourse analysis):ti,ab,kw

#4 #1 AND #2 AND #3

**JBI Database of Systematic Reviews**

**Abstract:** Peritoneal Dialysis OR Dialyses, Peritoneal OR Dialysis, Peritoneal OR Peritoneal Dialyses; Life Change Events OR event*, life change OR life change event OR life experience* OR experience*, life OR analysis, event history OR analyses, event history OR event history analyses OR event history analysis OR personal experience OR Perception OR Sensory Processing OR Processing, Sensory OR Attitude OR Attitudes OR Opinion*; Organizational Case Studies OR Case Studies, Organizational OR Studies, Organizational Case OR Management Case Studies OR Case Studies, Management OR Studies, Management Case OR Anthropology, Cultural OR Cultural Anthropology OR Material Culture* OR Culture, Material OR Ethnography OR Ethnographies OR grounded theory OR interview OR narrative OR phenomenology OR qualitative method OR qualitative research OR qualitative stud* OR thematic analysis OR focus group* OR descriptive Research OR discourse analysis

**China National Knowledge Infrastructure (CNKI)**

Subject: (( Peritoneal Dialysis OR Dialyses, Peritoneal OR Dialysis, Peritoneal OR Peritoneal Dialyses ) AND ( Life Change Events OR event*, life change OR life change event OR life experience* OR experience*, life OR analysis, event history OR analyses, event history OR event history analyses OR event history analysis OR personal experience OR Perception OR Sensory Processing OR Processing, Sensory OR Attitude OR Attitudes OR Opinion* ) AND (Organizational Case Studies OR Case Studies, Organizational OR Studies, Organizational Case OR Management Case Studies OR Case Studies, Management OR Studies, Management Case OR Anthropology, Cultural OR Cultural Anthropology OR Material Culture* OR Culture, Material OR Ethnography OR Ethnographies OR grounded theory OR interview OR narrative OR phenomenology OR qualitative method OR qualitative research OR qualitative stud* OR thematic analysis OR focus group* OR descriptive Research OR discourse analysis ))

**Chinese Biomedical Literature Database (CBM)**

Abstract: (( Peritoneal Dialysis OR Dialyses, Peritoneal OR Dialysis, Peritoneal OR Peritoneal Dialyses ) AND ( Life Change Events OR event*, life change OR life change event OR life experience* OR experience*, life OR analysis, event history OR analyses, event history OR event history analyses OR event history analysis OR personal experience OR Perception OR Sensory Processing OR Processing, Sensory OR Attitude OR Attitudes OR Opinion* ) AND (Organizational Case Studies OR Case Studies, Organizational OR Studies, Organizational Case OR Management Case Studies OR Case Studies, Management OR Studies, Management Case OR Anthropology, Cultural OR Cultural Anthropology OR Material Culture* OR Culture, Material OR Ethnography OR Ethnographies OR grounded theory OR interview OR narrative OR phenomenology OR qualitative method OR qualitative research OR qualitative stud* OR thematic analysis OR focus group* OR descriptive Research OR discourse analysis ))

**OpenGrey**

(“Peritoneal Dialysis” OR “Dialyses, Peritoneal” OR “Dialysis, Peritoneal” OR “Peritoneal Dialyses” ) AND ( “Life Change Events” OR “event*, life change” OR “life change event” OR “life experience*” OR “experience*, life” OR “analysis, event history” OR “analyses, event history” OR “event history analyses” OR “event history analysis” OR “personal experience” OR “Perception” OR “Sensory Processing” OR “Processing, Sensory” OR “Attitude” OR “Attitudes” OR “Opinion*” ) AND (“Organizational Case Studies” OR “Case Studies, Organizational” OR “Studies, Organizational Case” OR “Management Case Studies” OR “Case Studies, Management” OR “Studies, Management Case” OR “Anthropology, Cultural” OR “Cultural Anthropology” OR “Material Culture*” OR “Culture, Material” OR “Ethnography” OR “Ethnographies” OR “grounded theory” OR “interview” OR “narrative” OR “phenomenology” OR “qualitative method” OR “qualitative research” OR “qualitative stud*” OR “thematic analysis” OR “focus group*” OR “descriptive Research” OR “discourse analysis”)

**Deep Blue**

(“Peritoneal Dialysis” OR “Dialyses, Peritoneal” OR “Dialysis, Peritoneal” OR “Peritoneal Dialyses” ) AND ( “Life Change Events” OR “event*, life change” OR “life change event” OR “life experience*” OR “experience*, life” OR “analysis, event history” OR “analyses, event history” OR “event history analyses” OR “event history analysis” OR “personal experience” OR “Perception” OR “Sensory Processing” OR “Processing, Sensory” OR “Attitude” OR “Attitudes” OR “Opinion*” ) AND (“Organizational Case Studies” OR “Case Studies, Organizational” OR “Studies, Organizational Case” OR “Management Case Studies” OR “Case Studies, Management” OR “Studies, Management Case” OR “Anthropology, Cultural” OR “Cultural Anthropology” OR “Material Culture*” OR “Culture, Material” OR “Ethnography” OR “Ethnographies” OR “grounded theory” OR “interview” OR “narrative” OR “phenomenology” OR “qualitative method” OR “qualitative research” OR “qualitative stud*” OR “thematic analysis” OR “focus group*” OR “descriptive Research” OR “discourse analysis”)

**S1 Appendix search strategy**
