# Supplementary material for: The experiences of patients with peritoneal dialysis: A systematic review of qualitative evidence protocol
Source: PLoS One. 2023 Jul 19;18(7):e0288724. doi: 10.1371/journal.pone.0288724 (PMC10355407; doi:10.1371/journal.pone.0288724)
Supplement: Attachment — Submitted filename: Response to Reviewers.docx [file pone.0288724.s003.docx]

Response to Reviewers

Dear Editor and Reviewers,

First, we would like to thank you for your kind letter and for reviewers’ constructive comments concerning our artical. These comments are all valuable and helpful for improving our article. All the authors have seriously discussed about all these comments. According to the reviewers’ comments, we have modified our manuscript to meet with the requirements of your journal. In this revised version, changes to our manuscript with the document are highlighted by using red-colored text. Point-by-point responses to the reviewers are listed below in this letter.

Reviewer 1:

Comment to the author

*Regarding the manuscript, in the last paragraph on page 5, you stated, "The purpose of this study is to conduct a systematic review of qualitative research by synthesising the qualitative evidence from people with Parkinson's disease (PD)." Could you please verify whether the term "Parkinson's disease (PD)" has been used inadvertently? Your clarification would be much appreciated.*

Response:

We sincerely thank the reviewer for careful reading. As suggested by the reviewer, we have corrected the “Parkinson's disease” into “Peritoneal dialysis”.

Reviewer 2:

Comment 1:

*It can be considered if chronic PD results can be obtained in patients with hepatorenal syndrome and cardiorenal syndrome.*

Response 1:

This study focuses on the experiences of patients with end-stage renal disease treated with peritoneal dialysis to understand the internal beliefs, feelings and perceptions of peritoneal dialysis treatment in this disease population. This will provide a theoretical basis for researchers and healthcare professionals to gain a deeper and more comprehensive understanding of this population and to provide targeted interventions to improve patient outcomes. All patients undergoing peritoneal dialysis who met the inclusion and exclusion criteria will be included in this study without regard to primary medical conditions

Comment 2:

*Towards the end of page 5, it is referred to as parkindon disease for PD abbreviation.... let's fix it.*

Response 2:

We feel sorry for our carelessness. In our resubmitted manuscript, the typo is revised. Thanks for your correction.

Comment 3:

*In my opinion, the review plan is acceptable after correcting the native English language.*

Response 3:

We tried our best to improve the manuscript and made some changes to the manuscript. These changes will not influence the content and framework of the paper. And here we did not list the changes but marked in red in the revised paper. We appreciate for Editors/Reviewers’ warm work earnestly and hope that the correction will meet with approval.
